# Effect of vitamin K2 in the treatment of nocturnal leg cramps in the older population: Study protocol of a randomized, double-blind, controlled trial

**DOI:** 10.3389/fnut.2023.1119233

**Published:** 2023-02-23

**Authors:** Ying Li, Rui Zhu, Li Wang, Jing Tan

**Affiliations:** ^1^Department of Hematology, Chengdu Third People's Hospital, Chengdu, Sichuan, China; ^2^School of Clinical Medicine, North Sichuan Medical College, Nanchong, Sichuan, China; ^3^Department of Neurology, Affiliated Hospital of North Sichuan Medical College, Nanchong, Sichuan, China

**Keywords:** vitamin K2, nocturnal leg cramps, older population, muscle cramps, randomized controlled trial

## Abstract

**Introduction:**

Nocturnal leg cramps (NLCs) are sudden contractions of the leg muscles, usually in the posterior calf muscles at night, affecting sleep quality. Because the precise pathophysiology of NCLs is unclear, different interventions have been proposed. There is conflicting evidence regarding the efficacy of conventional interventions in preventing cramps. Thus, the present study aims to investigate the effects of vitamin K2 for NLCs in a prospective randomized, double-blind, controlled trial.

**Methods and analysis:**

This multicenter, randomized, double-blind, placebo-controlled clinical study will enroll older age (≥65-year-old) with two or more documented episodes of NLCs during 2 weeks of screening. Participants will be randomized to receive vitamin K2 or a similar-looking placebo for 8 weeks in a 1:1 ratio. Follow-up visits will be scheduled each week at the beginning of 4-week intervention, then participants will be visited semimonthly. The primary outcome is the difference in the mean number of NLCs per week in the vitamin K2 and placebo arms. The secondary outcomes include the severity and duration of NLCs in the vitamin K2 and placebo arms. Two hundred patients will be needed, for this two-treatment parallel design study, to achieve a probability is 90% that the study will detect a treatment difference at a two-sided 0.04 significance level, if the difference between treatments is 3.6 (difference in means between treatment arms) NLC events.

**Discussion:**

Nocturnal Leg Cramps (NLCs) are a common musculoskeletal disorder in the general population, but effective and safe interventions have not been established. Our previous study has shown vitamin K2 was effective to reduce the frequency, severity, and duration of dialysis-related muscle cramps with a good safety profile. This randomized controlled trial (RCT) of rigorous methodological design will help to establish the effectiveness of vitamin K2 for the management of NLCs in older population. The findings of this RCT will encourage the studies of vitamin K2 in musculoskeletal disorders.

**Clinical Trial Registration:**

www.ClinicalTrials.gov, identifier, NCT05547750.

## 1. Introduction

Nocturnal leg cramps (NLCs) are spontaneous contractions of muscles. The gastrocnemius is commonly involved (1), lasting from a few seconds to a few minutes (2). Patients might wake up with pain during attacks, making it difficult to sleep for a short period. It commonly occurs >60-year-old (3). The medical history and physical examination are usually sufficient to differentiate nocturnal leg cramps from other conditions, such as restless legs syndrome, claudication, myositis, and peripheral neuropathy. Factors that may lead to leg cramps attacks include hemodialysis, electrolyte imbalance, metabolic disorders, and congenital disorders (4). The cramps can be relieved by passive stretching of the gastrocnemius and deep tissue massage, but such prevention is limited, especially in patients with refractory muscle cramps (5). Quinine has been shown to be effective in treating NLCs but is not recommended by the US Food and Drug Administration due to severe side effects (6). Magnesium supplements are often used as a preventative treatment for NLCs (7, 8); however, their effectiveness is controversial (2, 9, 10). Magnesium supplements are widely marketed for the prophylaxis of NLCs since a double-blind, placebo-controlled study proved their effectiveness in pregnant women (11). However, magnesium administration did not show significant benefits in NLCs in double-blind, placebo-controlled studies (12, 13). Meta-analysis of some randomized control trials (RCTs) showed that magnesium therapy did not appear to be effective in the treatment of NLCs in the general population, but may have a negligible effect in pregnant women (14). Therefore, seeking new approaches to manage NLCs is imperative.

Vitamin K is a fat-soluble vitamin involved in carboxylation and activating several dependent proteins. It is found in two isoforms (phylloquinone (vitamin K1) and menaquinone (vitamin K2)) that differ in length and degree of saturation of the side chain. In addition to their role in coagulation, vitamin K-dependent proteins are involved in vascular calcification and osteoporosis physiology. Accumulating evidence has shown the beneficial effects of vitamin K2 supplementation on cardiovascular and bone health (15).

Another study revealed that vitamin K3 relieved muscle cramps by effectuating the voltage-dependent calcium channels to release the calcium stored in the cells, thus reducing the frequency of muscular contractions (16). To the best of our knowledge, no study has yet investigated the efficacy of vitamin K in NLCs. In addition, vitamin K2 has a good safety profile compared to other medications. Our pilot study demonstrated that vitamin K2 supplementation decreases the frequency, duration, and severity of muscle cramps in hemodialysis patients (17). To further investigate the efficacy and safety of vitamin K2 in NLCs, we designed this prospective, multicenter, randomized, double-blind trial.

## 2. Methods and analysis

### 2.1. Objective

The objective of the present study is to evaluate the efficacy and safety of vitamin K2 in the older population with NLCs.

### 2.2. Trial design

This prospective, multicenter RCT will recruit participants from two tertiary hospitals, Chengdu Third People’s Hospital and Affiliated Hospital of North Sichuan Medical College, with the diagnosis of NLCs. This manuscript is according to the Standard Protocol Items: Recommendations for Interventional Trials (SPIRIT) guidelines (18).

The study will conduct as a randomized controlled, double-blinded trial. The subjects screened with NLCs will be randomly assigned to two arms: the vitamin K2 arm (vitamin K2 180 μg/day) and the placebo arm. The arms would consist of an equal number of participants, and the study would be conducted double-blind between the participants and the researchers during observation. The overall framework is to compare the treatment outcomes between the two groups.

**Figure 1:**
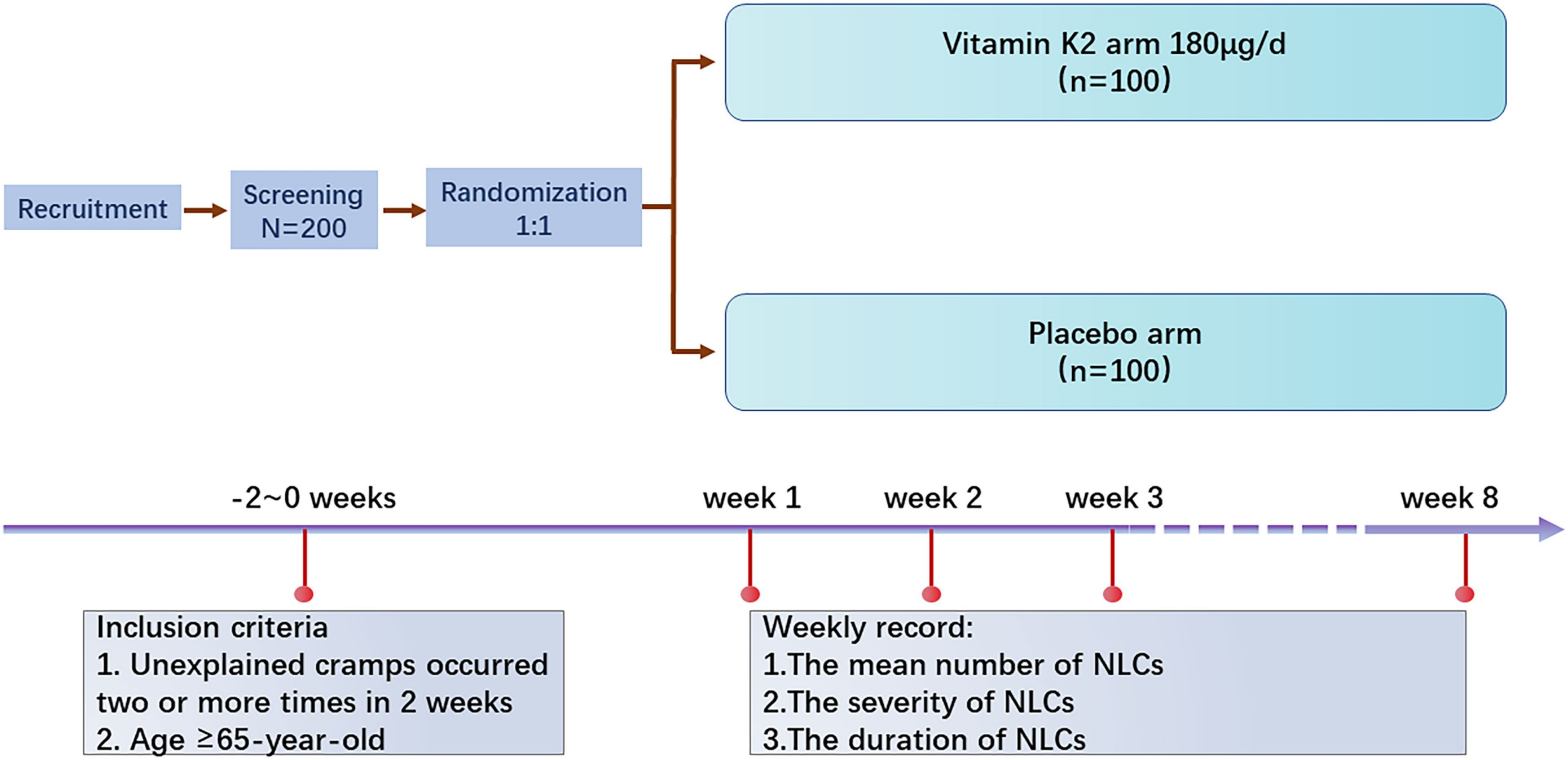
Study design and flow chart.

A total of 200 participants will be randomly assigned to the two study groups using a 1:1 randomization protocol (*n* = 100/group). The vitamin K2 arm takes vitamin K2 180 μg/day at bedtime and the placebo arm takes a placebo at bedtime for 8 weeks. The frequency of muscle cramps and the duration and severity of each attack in both arms will be recorded every week (Figure 1).

Medical evaluation and enrolment procedure. All participants will be recruited through recruitment advertisement from September 2022 to September 2023. Potential participants suffering from NLCs are willing to participate in this study. In that case, they may contact the research assistant, who will make a medical history interview to screen the participants. Eligible participants will be invited to participate in a physical examination to confirm NLC diagnosis and assess eligibility for participation in the study.

### 2.3. Inclusion criteria

1. Unexplained cramps occurred two or more times in 2 weeks.
2. Age ≥ 65-year-old.

### 2.4. Exclusion criteria

1. Cramps caused by specific metabolic diseases and specific neuropathies (hypothyroidism, hemodialysis, hypoglycemia, alcoholism, amyotrophic lateral sclerosis, poliomyelitis complications, lumbar spinal stenosis, Parkinson’s disease, radiculopathies, and motor neuron diseases).
2. Suffering from malignant tumors (breast cancer, prostate cancer, lymphoma, and multiple myeloma).
3. Taking diuretics, or vitamin K antagonist.
4. Taking supplements with vitamin K2 within 2 months before enrollment.

A research assistant will meet with the eligible participants after the medical evaluation and obtain their written informed consent. All participants’ demographic variables, such as age, sex, medical history, and lifestyle (smoking and alcohol use), will be collected before intervention (baseline). Participants will also be asked relevant questions about the duration of symptoms and previous treatments.

Randomization and blinding participants will be classified into two intervention groups at a ratio of 1:1, using a computer-generated randomized sequence with varying unknown block sizes for all study centers without stratification. A research assistant not involved in clinical care and participant evaluations will prepare sequentially numbered, opaque, sealed envelopes based on a random list and ensure that anyone will not access or influence the allocation data. The participants, outcome assessor, and statistician will be blinded to group allocation and not involved in the treatment procedures.

## 3. Intervention description

### 3.1. Vitamin K2

Vitamin K2 is a fat-soluble and one of the body’s indispensable vitamins. It is mainly synthesized by gut bacteria in the body and plays a role in the mitochondrial electron transport chain (19). It boosts calcium metabolism, acts on osteoblasts, and promotes bone tissue calcification. It also inhibits osteoclasts from causing bone resorption, thus increasing bone density, and preventing osteoporosis (20). Furthermore, it regulates the use of calcium and promotes the inhibition of vascular calcification by matrix Gla protein (MGP) activity (21). Vitamin K2 supplementation at recommended dosage does not affect vitamin K-dependent coagulation factors’ activity. It does not enhance the carboxylation of prothrombin in healthy individuals. Vitamin K2 administration does not alter the hemostatic balance in healthy populations without anticoagulation treatment. Thus, vitamin K2 is deemed to have a good safety profile (22).

### 3.2. Placebo

Placebo tablets appear similar to those of vitamin K2, and the participants are unaware whether they are assigned to the vitamin K2 or the placebo group.

Criteria for trial termination or withdrawal participants may withdraw from the trial voluntarily for personal reasons or terminate it if they become unwell due to the treatment. During the experiment, researchers will record all adverse events.

## 4. Data management

Data will be collected at baseline and every week after random assignment (Table 1). Phone calls from research assistants will be programmed each week to maximize participant compliance in subsequent assessments. A registered participant will be excluded from the study if exclusion criteria are detected after registration. Researchers will record the cause and date of suspension. The consent to use data collected before the participant’s withdrawal will be included in the informed consent form. We will perform all data analyses according to the intention to treat principle, and the analysis, data collection, and processing will be blinded with respect to treatment group assignment. Randomized participants who do not complete the study will be included in their assigned study groups for the primary analysis.

**Table 1:** Study evaluation procedures and timeline.

Trail phase	Screening −2 week	Baseline 0 week	Intervention							
			1 week	2 week	3 week	4 week	5 week	6 week	7 week	8 week
Sign the informed consent form	×									
Determine eligibility	×									
Obtain medical and demographic data		×								
Fill in the general information	×									
Comorbidities and treatment			×	×	×	×	×	×	×	×
Outcome measures										
The mean number of NLCs			×	×	×	×	×	×	×	×
The severity of NLCs			×	×	×	×	×	×	×	×
The duration of NLCs			×	×	×	×	×	×	×	×
Physical examination						×				×

## 5. Primary endpoint

The mean number of NLCs attacks per week (During the 8-week investigation, the differences in the frequency of attacks will be recorded and compared between vitamin K2 and placebo arms).

## 6. Key secondary endpoints

1. Duration of muscle cramps in minutes (During the 8-week investigation, the differences in the duration of attacks will be recorded and compared between vitamin K2 and placebo arms).
2. The severity of muscle cramps using a 1–10 analog scale (During the 8-week investigation, pain severity during attacks will be recorded and compared between vitamin K2 and placebo arms). The participants will be asked to record the severity of cramping with a 1–10 analog scale. That is, 1–3 points, mild pain, tolerable, does not affect sleep; 4–6 points, moderate pain, affects sleep, also tolerable; 7–10 points, sharp pain, intolerable.

## 7. Adverse events

All adverse events, defined as negative or unwanted reactions to intervention, will be recorded based on the symptoms reported by the patients and observations by a researcher at every visit. Follow-up visits will be scheduled each week in the beginning of 4-week intervention, then participants will be visited semimonthly of adverse events monitoring.

## 8. Sample size

The difference in the number of episodes of NLCs during the treatment period of 8 weeks, will serve as the sole primary efficacy endpoint for this study. We calculated that a total of 200 patients will be needed, for this two-treatment parallel design study, to achieve a probability is 90 percent that the study will detect a treatment difference at a two-sided 0.04 significance level, if the true difference between treatments is 3.6 (difference in means between treatment groups) NLC events. This is based on the assumption that the standard deviation of the mean number of NLC events is 8.

## 9. Statistical methods

SPSS statistical software is used for data analysis. Non-parametric tests (Mann–Whitney U test) are used for non-normal data. Results are given as medians with 95% confidence intervals (CI). Approximately normally distributed data are described as means and standard deviations. Other statistical tests used include Student’s t-tests for continuous normally distributed data and Chi-Square tests for categorical data are stated with the results.

## 10. Quality assurance/monitoring/management

In order to standardize the procedures of staff training and learning, such as participant recruitment, outcome measures, data import, security, and management and analysis, a manual of operations and procedures and a case report form will be developed, including the monitoring plans to assure participant protection and data integrity, thus facilitating consistency in protocol implementation and data collection. The investigators, physicians, research assistants, outcome assessors, and statisticians should be trained in good clinical practice. The trained project managers will visit each center for monitoring to ensure data quality and compliance with the trial protocol.

All data will be stored electronically and strictly in a database with secure and restricted access. Encryption will be used for data transmission, and any information identifying individuals will be removed. Data will only be de-identified for analysis after this study.

## 11. Discussion

NLCs are a common cause of sleep disturbance among the older population. Although experienced by most people at some point in life, there is little concern about leg cramps because of their sporadic and infrequent occurrence. However, in patients with frequent NLCs, an annoying physical symptom may cause significant distress and nighttime disturbance. Forceful stretching is often the most straightforward remedy. Nonpharmacological methods of preventing leg cramps have not been proven effective for most people (2). In terms of pharmacological treatments, quinine (23) and magnesium (2, 7, 9, 10, 13) have been studied extensively; however, the results on the exact efficacy remain controversial. Other medications, such as calcium channel blockers, vitamin E, vitamin B complex, and antiepileptic medications (6, 24, 25), might alleviate NLCs, but their efficacy is not yet defined due to the low methodological quality of the trials.

Previously, we demonstrated that vitamin K2 supplementation decreases the frequency, duration, and severity of muscle cramps and is safe for hemodialysis patients (17).

NLC diagnosis criteria is based on subjective report without objective evaluation and parameters, which poses a limitation to this study.

To the best of our knowledge, this is the first to investigate the efficacy and safety of vitamin K2 in the treatment and prevention NLCs. The findings of this RCT will encourage further studies of vitamin K2 in musculoskeletal disorder.

## Ethics statement

The studies involving human participants were reviewed and approved by the Ethics Committee of the Third People’s Hospital of Chengdu (approval No. 2022-S17). Written informed consent to participate in this study was provided by the participants.

## Author contributions

JT designed the study. JT, LW, YL, and RZ completed the research ethics registration and were responsible for the observation of patients in hospital. JT and YL drafted and submitted the manuscript. All authors contributed to the article and approved the submitted version.

## Funding

This work was supported by the China Health Promotion Foundation. This study was a non-profit academic clinical research study.

## Conflict of interest

The authors declare that the research was conducted in the absence of any commercial or financial relationships that could be construed as a potential conflict of interest.

## Publisher’s note

All claims expressed in this article are solely those of the authors and do not necessarily represent those of their affiliated organizations, or those of the publisher, the editors and the reviewers. Any product that may be evaluated in this article, or claim that may be made by its manufacturer, is not guaranteed or endorsed by the publisher.
